# The comparison of the performance of four whole genome amplification kits on ion proton platform in copy number variation detection

**DOI:** 10.1042/BSR20170252

**Published:** 2017-07-07

**Authors:** Xinyi Zhang, Bo Liang, Xiaoyan Xu, Feifei Zhou, Lingyin Kong, Jingjing Shen, Yingying Xia, Liming Xuan, Yan Mao, Yongfeng Xue, Caixia Liu, Jichun Tan

**Affiliations:** 1 Reproductive Medical Center of Gynecology and Obstetrics Department, Shengjing Hospital Affiliated to China Medical University, Shenyang, Liaoning 110004, China; 2 Basecare Medical Device Co., Ltd., Suzhou, Jiangsu 215000, China; 3 State Key Laboratory of Microbial Metabolism, Joint International Research Laboratory of Metabolic and Developmental Sciences, School of Life Sciences and Biotechnology, Shanghai Jiao Tong University, 800 Dongchuan Road, Shanghai 200240, China.; 4 Cyagen Health Inc., Suzhou, Jiangsu 215400, China; 5 Key Laboratory of Maternal-Fetal Medicine of Liaoning Province, Shengjing Hospital Affiliated to China Medical University, Shenyang, Liaoning 110004, China.; 6 Key Laboratory of Obstetrics and Gynecology of Higher Education of Liaoning Province, Shengjing Hospital Affiliated to China Medical University, Shenyang, Liaoning 110004, China.

**Keywords:** CNV, Ion Proton, single-cell sequencing, WGA

## Abstract

With the development and clinical application of genomics, more and more concern is focused on single-cell sequencing. In the process of single-cell sequencing, whole genome amplification is a key step to enrich sample DNA. Previous studies have compared the performance of different whole genome amplification (WGA) strategies on Illumina sequencing platforms, but there is no related research aimed at Ion Proton platform, which is also a popular next-generation sequencing platform. Here by amplifying cells from six cell lines with different karyotypes, we estimated the data features of four common commercial WGA kits (PicoPLEX WGA Kit, GenomePlex Single Cell Whole Genome Amplification Kit, MALBAC Single Cell Whole Genome Amplification Kit, and REPLI-g Single Cell Kit), including median absolute pairwise difference, uniformity, reproducibility, and fidelity, and examined their performance of copy number variation detection. The results showed that both MALBAC and PicoPLEX could yield high-quality data and had high reproducibility and fidelity; and as for uniformity, PicoPLEX was slightly superior to MALBAC.

## Introduction

With the decreasing cost and increasing clinical application of next-generation sequencing (NGS), more and more concerns are focused on NGS. Ion Proton is a semiconductor-based NGS platform [1]. The reads yielded by Ion Proton platform are length-variable single-end reads [2], which is quite different from those of Illumina sequencing platforms, e.g. Hiseq series [3,4]. Ion Proton is one of the currently main NGS platforms and is widely applied to diverse fields, including exome sequencing, transcriptome sequencing, single-cell sequencing etc. [5–9], and especially it often occurs in clinical examination, such as noninvasive prenatal diagnosis [10] and preimplantation genetic screening [11].

Single-cell sequencing offers cellular-level genetic information, so it is an important approach to study early embryo development, neuron genomic heterogeneity, carcinogenesis, and uncultivable bacteria etc. [12,13]. Along the process of single-cell sequencing, whole genome amplification (WGA) is a key step [14], because of the low quantity of available materials. There are plenty of commercial WGA kits and different kits are based on different strategies: multiple displacement amplification (MDA), degenerate-oligonucleotide-primed PCR (DOP-PCR), and multiple annealing and looping-based amplification cycles (MALBAC, MALBAC-like) are three major WGA strategies. Different strategy may cause different WGA performance and some studies have paid efforts in the comparison of WGA methods [15–19]. However, current related researches are conducted on Illumina sequencing platforms, the results have not validated on Ion Proton platform.

To address this issue, we tested four common commercial WGA kits—PicoPLEX WGA Kit (which we called PicoPLEX, Rubicon Genomics, U.S.A.), GenomePlex Single Cell Whole Genome Amplification Kit (GenomePlex, Sigma–Aldrich, U.S.A.), MALBAC Single Cell WGA Kit (MALBAC, Yikon Genomics, China), and REPLI-g Single Cell Kit (MDA, Qiagen, Germany). The first two kits were based on DOP-PCR method, and the two following were based on MALBAC and MDA respectively. The amplified products were sequenced on Ion Proton platform, and then we evaluated the data features, compared the difference among different sample types, and examined their performance in copy number variation (CNV) detection. In this way, we found that MALBAC and PicoPLEX could yield high-quality data and exhibited high reproducibility, and in terms of uniformity, PicoPLEX was slightly better than MALBAC. Our results provided system comparison among the four common WGA kits and guidance for future studies related to single-cell sequencing on Ion Proton platform.

## Materials and methods

### Samples

Six cell lines in the study were bought from the Coriell Institute (Coriell Biorepositories, U.S.A.), and their karyotypes covered common CNV types, including four aneuploids (+13, +18, +21, and XO), one microdeletion (del(16)(pter->p12::p11.2->qter)), and one unbalanced translocation (der(4)t(4;12)(p16.1;p12)pat). Single-cell, multi-cell, and genomic DNA (gDNA) samples were separately prepared for the six cell lines.

Single cell and multi cells (3–5 cells) were isolated by micromanipulating under a dissection microscope (Olympus SZ61, Japan) using a finely pulled glass Pasteur pipette. Then, the isolated cells were washed twice with phosphate-buffered saline (PBS) and transferred into 200 μl PCR tubes containing 2.5 μl of PBS.

The gDNAs of the cell lines were extracted by Qiagen DNeasy Blood and Tissue Kit (Qiagen, Germany) according to the manufacturer’s instructions. The concentration of purified gDNA was determined using Quant-iT Assay Kit with Qubit fluorometer (Invitrogen, U.S.A.) and purity analysis was conducted by Nanodrop 2000 (Thermo Scientific, Germany). Then, 6 ng of gDNA was extracted and serially diluted 1000-fold by Nuclease-Free Water (Qiagen, Germany) to adjust the concentration to 6 pg/μl. We aliquoted 2.5 μl of the diluted solution so that each aliquot contained 15 pg of gDNA, which was equivalent to the amount of the DNA in a single cell (∼6.6 pg of DNA), taking into account the random loss of DNA due to limiting dilution.

These samples were stored in −80°C before WGA. All procedures were performed in a dedicated laboratory with positive pressure HEPA-filtered air to avoid contamination.

### Whole genome amplification

According to the recommended protocols, Single-cell, multi-cell, and gDNA samples were amplified by four common commercial WGA kits: PicoPLEX WGA Kit (Rubicon Genomics, U.S.A., version: R30050), GenomePlex Single Cell Whole Genome Amplification Kit (Sigma–Aldrich, U.S.A., version: WGA4-50RXN), MALBAC Single Cell WGA Kit (Yikon Genomics, China, version: YK001B), and REPLI-g Single Cell Kit (Qiagen, Germany, version: 150343). After WGA, the products were purified by Agencourt Ampure XP beads (Beckman Coulter, U.S.A.). The purified DNA was quantified using the Quant-iT Assay Kit with Qubit fluorometer (Invitrogen, U.S.A.), and the purity was determined by Nanodrop 2000 (Thermo Scientific, U.S.A.). Then the amplified products were separated by 2% agarose gels electrophoresis in TBE buffer (90 mmol/l Tris-borate, pH 8.0, 2 mmol/l EDTA), stained by SYBR Green (Invitrogen, U.S.A.), and visualized by Image Master software (Bio-Rad, U.S.A.).

### Library construction and whole-genome DNA sequencing

The WGA products were fragmented to an average size of 170 bp and end ligated with sequencing adaptors carrying identifiable barcode for Ion Proton platform. Tagged fragments were then amplified by PCR with adaptor primers to generate 250 bp (80 bp adaptors/barcode and 170 bp insert DNA) sequencing libraries. Finally, the libraries with different barcode were pooled together and sequenced on Ion Proton platform with PI chip.

### Data analysis

All sequencing data were aligned to the human genome reference sequences (version: NCBI Build37/hg19) by TMAP software. Then, PCR duplications were removed by Picard software, and uniquely mapped reads, reads with mapping quality score (MAPQ) ≥10 and length ≥35 bp, were kept for the following analysis.

For each sample, all chromosomes were divided into segments of the same size (20 kbp), called windows. The number of uniquely mapped reads and GC content in each window was determined. Then, we filtered windows containing no reads and windows with ‘N’s in the reference sequences. The number of uniquely mapped reads in the remaining windows was corrected and normalized based on their GC content by LOESS regression. Finally, taking corresponding samples from normal humans’ oral epithelium as control, we applied the circular binary segmentation (CBS) algorithm to segment the genome into regions with equal copy number, and screened out CNVs whose mean of the log of normalized read count were less than −0.51 or more than 0.37 and size were more than 4 Mbp.

## Results

### The yield and size of WGA products

**Figure 1:**
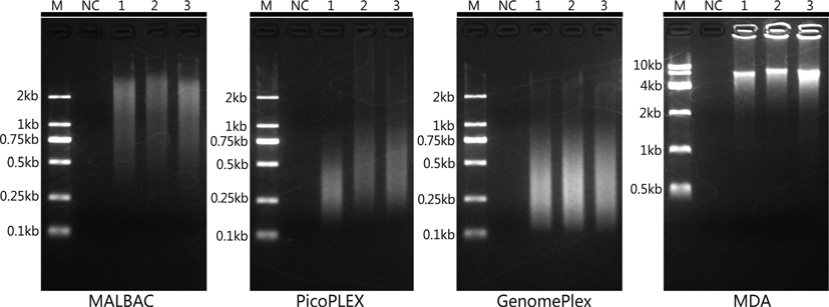
Gel electrophoresis of WGA products M, DNA molecular weight markers; NC, negative control; Lane 1, amplified gDNA sample; Lane 2, single-cell sample; Lane 3, multi-cell sample.

Single-cell, multi-cell, and gDNA samples were amplified by MALBAC, PicoPLEX, GenomePlex, and MDA respectively. Gel analysis was used to determine the yield and size range of the WGA products (Figure 1). Among different sample types, the yield and size range were similar in the same WGA kit. And the yield was 1.0 ± 0.32 μg in MALBAC, 2.0 ± 0.19 μg in PicoPLEX, 4.4 ± 2.3 μg in GenomePlex, and 13.6 ± 6.7 μg in MDA; the size of products distributed approximately 0.5–2.0 kb in MALBAC, 0.25–1.0 kb in PicoPLEX, 0.1–1.0 kb in GenomePlex and 4.0–10.0 kb in MDA. Above results were consistent with the results of previous studies [16,20].

### Quality assessment of sequencing data

**Figure 2:**
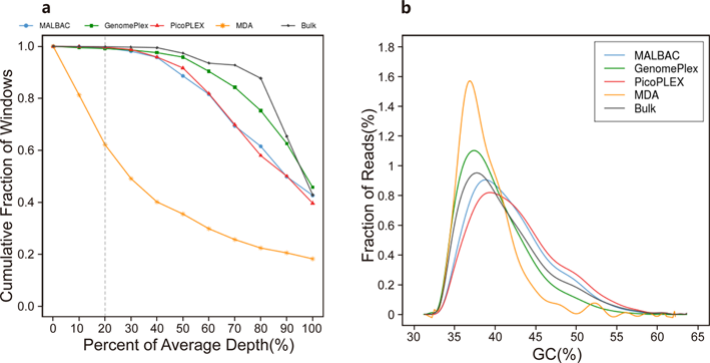
Uniformity of coverage and GC bias analysis (**a**) The cumulative distribution of read depth. The cumulative fraction of windows on the depth of 20% of average depth (dash line) is a metric for data quality assessment. (**b**) The reads distribution under different GC content levels.

In order to estimate the quality of data generated from the four kits on Ion Proton platform, we evaluated the quantity of raw reads, depth, coverage, mapping rate, duplication rate, the ratio of unique reads, genomic GC percent, and median absolute pairwise difference (MAPD) (Table 1 and Figure 2). The results showed that although the means of GC percents of the four kits were almost the same, the peaks of GC percents of MALBAC and PicoPLEX slightly shifted to the right comparing with the bulk data, while those of MDA and GenomePlex were highly consistent with the bulk data (Figure 2b). With similar number of raw reads to the other three kits, the coverage of MDA was the lowest among the four kits and the coverage on different depths suggested that the uniformity of depth in MDA was much worse than the other three kits and deviated severely from the bulk data. According to Lander et al. [21] and our practical experience, we regarded sequencing data whose cumulative coverage on 20% of the average depth was less than 90% as not quantitated data for further analysis. In our results, the cumulative coverage on 20% of the average depth of MDA was only 62%, while the other three kits were ∼99%.

**Table 1:** Basic statistics of single cells amplified by different kits

Reagent	Raw reads	Depth	Coverage (%)	Mapping rate (%)	Duplication rate (%)	Unique reads ratio (%)	GC (%)	MAPD
MALBAC	5,244,767	0.26	15.82	98.05	6.98	58.11	40.91	0.31
MDA	4,699,604	0.31	8.06	99.21	10.26	55.46	40.46	2.48
GenomePlex	4,768,010	0.23	13.72	97.63	10.06	40.95	40.90	0.42
PicoPLEX	5,791,391	0.30	15.37	97.20	11.33	39.19	40.92	0.24
Bulk	4,644,129	0.25	18.71	98.79	9.61	57.34	40.91	0.20

To assess copy number (CN) noise across the whole genome, we employed MAPD algorithm [22], which was widely applied in quality assessment for microarray data and also was adapted on Hiseq platform [23,24]. MAPD algorithm was the median of the absolute values of deviation between the log2 CN ratios of every pair of adjacent windows. Theoretically, higher MAPD scores reflected greater genomic noise and we eliminated data whose MAPD score was higher than 0.4.

**Figure 3:**
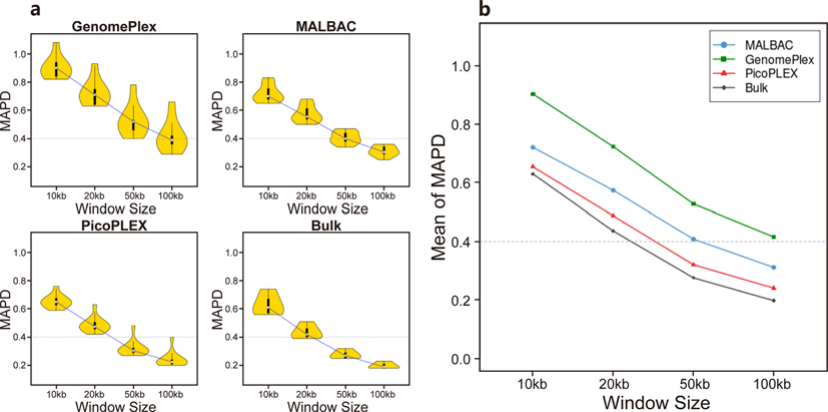
MAPDs of WGA kits and bulk data in different window sizes (**a**) MAPDs of read count yielded by GenomePlex, MALBAC, PicoPLEX, and the bulk-cell sample respectively, are compared in different window sizes. The threshold of MAPD is 0.4 (dash line). (**b**) The average MAPDs among all samples of GenomePlex, MALBAC, PicoPLEX, and the bulk data respectively, are plotted against window sizes.

We tested the performance of data in different window sizes (Figure 3) and found that larger window size contained less genomic noise, at the cost of CNV resolution. MAPDs in 100kb windows of both PicoPLEX and MALBAC passed the threshold (0.23 ± 0.05 and 0.31 ± 0.04 respectively), and the mean of MAPD of PicoPLEX was lower and closest to that of the bulk data in the four kits. MAPD of MDA was as high as 2.48 even in 100 kb windows, consistent with the bad uniformity of coverage with depth in MDA.

### Uniformity and reproducibility analysis

**Figure 4:**
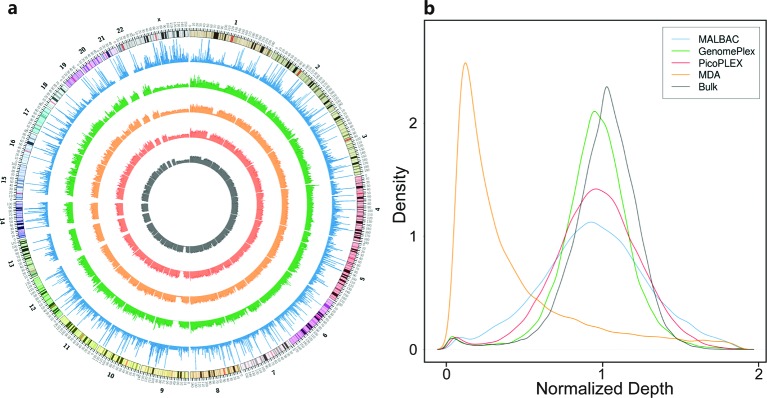
Uniformity of read depth across the whole genome (**a**) Read depth distribution across the whole genome. The five circles from inside to outside are normalized read depth in 100 kb windows of the bulk data (black), GenomePlex (light red), PicoPLEX (orange), MALBAC (green), and MDA (blue). The circle outside them is the chromosomal coordinate in a pter-qter orientation, which is zoomed out by 1,000,000 times, i.e. the actual position is the number on the circle multiplying 1 Mbp. (**b**) The Kernal density plots of normalized read depth in 100 kb windows of the four WGA kits and the bulk data.

A uniform distribution of read depth was the basis of CNV analysis. Among the four kits, the narrow distribution of read depth approximately 1 in GenomePlex and PicoPLEX suggested their high uniformity, while the distribution of MALBAC was wider and had fat tails, indicating relatively lower uniformity in MALBAC. The distribution of MDA severely deviated from 1 and the distributions of the other three WGA kits and the bulk data, supporting the conclusion mentioned above (Figure 4).

**Figure 5:**
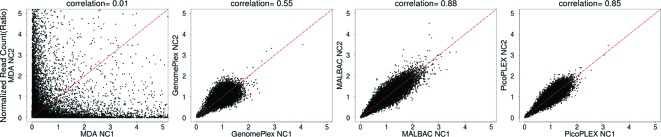
Amplification reproducibility of WGA kits The normalized read count in 100 kb windows of WGA products is plotted against that of another repeat of the same WGA kit, and the Pearson correlation coefficient between the two repeats is showed on the top. From left to right are plots of MDA, GenomePlex, MALBAC, and PicoPLEX.

To estimate the reproducibility of different kits, we compared the normalized read ratios between two repeats of the four kits and calculated their Pearson correlation coefficient (Figure 5). The correlation coefficients between two repeats of MALBAC and PicoPLEX were 0.88 and 0.85 respectively, while that of GenomePlex was only 0.55. MDA showed the lowest correlation coefficients, i.e. 0.01, indicating that there was nearly no linear correlation between two repeats of MDA.

In summary, GenomePlex products had high uniformity but low reproducibility and MALBAC had high reproducibility but lower uniformity than GenomePlex and PicoPLEX, while MDA had bad uniformity and bad reproducibility. PicoPLEX was superior to the other three kits in both uniformity and reproducibility, and was recommended for CNV analysis on Ion Proton platform.

### Comparison among different types of samples

**Figure 6:**
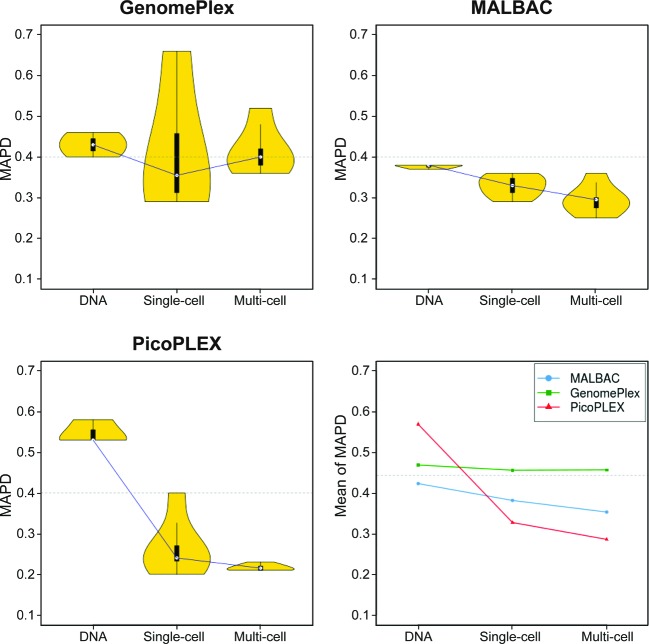
MAPDs of WGA kits in different types of samples MAPDs of read count yielded by GenomePlex, MALBAC, and PicoPLEX respectively, are compared in different sample types.

To examine the performance of different kits in different types of samples, we extracted gDNA, single cell, and multi cells from normal humans’ oral epithelium and conducted whole genome amplification on all of them by GenomePlex, MALBAC, and PicoPLEX (MDA was excluded because its performance was not suitable for CNV analysis as mentioned above), and then sequenced them on Ion Proton platform. Except for GenomePlex whose MAPDs fluctuated around 0.40, the MAPD of MALBAC and PicoPLEX decreased from gDNA to multi-cell samples (Figure 6), indicating a lower genomic noise level in multi-cell samples.

**Figure 7:**
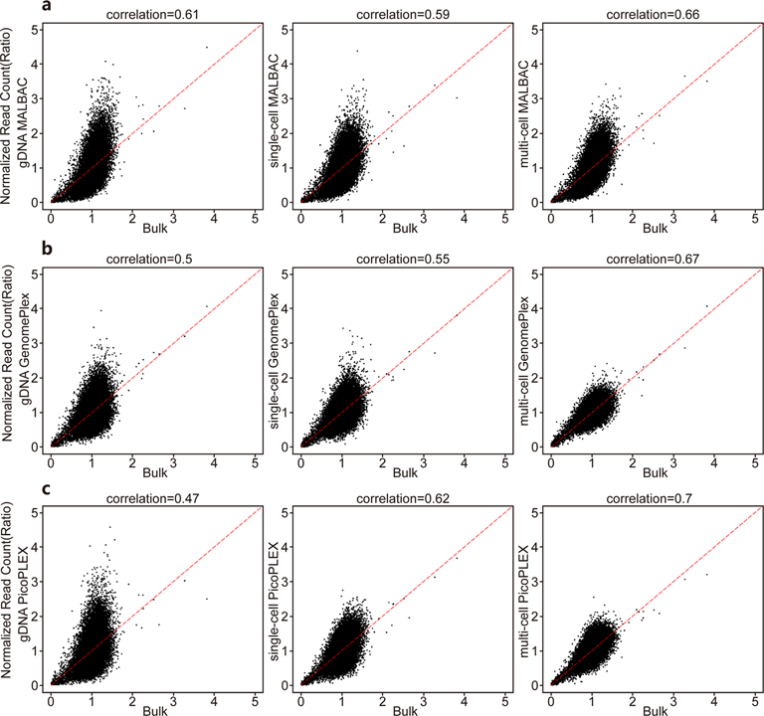
Amplification bias of WGA kits in different types of samples The normalized read count of WGA products of (**a**) MALBAC, (**b**) GenomePlex, and (**c**) PicoPLEX in different sample types is plotted against that of the unamplified sample. From left to right are plots of gDNA samples, single-cell samples, and multi-cell samples respectively.

In order to evaluate the amplification bias of different WGA kits in different sample types, we plotted the normalized read count of samples amplified by different WGA kits against the unamplified one (Figure 7). The correlation coefficients between samples amplified by MALBAC and the bulk-cell sample fluctuated around 0.62 among different sample types, and the correlation coefficients of GenomePlex and PicoPLEX demonstrated higher consistency with unamplified data in multi-cell samples than in gDNA or single-cell samples, indicating smaller amplification bias in multi-cell samples. In total, PicoPLEX had the greatest correlation coefficients except for gDNA samples, suggesting it contained less amplification bias than others, i.e. PicoPLEX showed the best fidelity.

### CNVs detection

**Figure 8:**
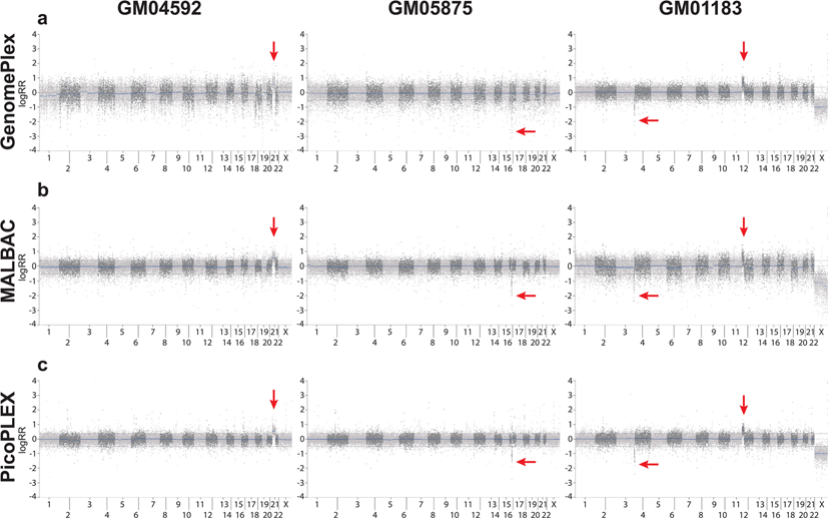
Plots of copy number of samples from different cell lines amplified by different WGA kits The log of normalized read count (logRR) of WGA products of (**a**) GenomePlex, (**b**) MALBAC, and (**c**) PicoPLEX is plotted. From left to right are plots of samples from cell lines of aneuploids (GM04592), microdeletion (GM05875), and unbalanced translocation (GM01183) respectively. The *x*-axis of each plot stands for 23 chromosomes of human without chromosome Y, and points of adjacent chromosomes are drawn in different colors. Segments marked by red arrows are confirmed CNVs in the cell line. Because the control consists of female samples while the gender of GM01183 is male, the copy number of chromosome X in GM01183 appears less than normal.

To estimate the effect of the four kits on variation detection, we selected cell lines of six different CNVs with confirmed karyotypes, including four aneuploids, one microdeletion, and one unbalanced translocation, and detected CNVs in their multi-cell samples of the four kits. Using the same software and parameters, the four WGA kits showed different performance in CNV detection (Table 2 and Figure 8). The CNVs detected in PicoPLEX and MALBAC products were completely consistent with the confirmed CNVs of each cell line, while there were two false positives results in GenomePlex. The detected CNV results were totally wrong in MDA under the same condition, associating with its bad genomic uniformity of depth.

**Table 2:** CNVs detection results of six confirmed cell lines

Cell Line	Confirmed CNV	MALBAC	PicoPLEX	GenomePlex	MDA
GM00857	45,XO	**45,XO**	**45,XO**	**45,XO**	+21, −22
GM05875	46,XX, del(16)(pter ->p12::p11.2->qter)	**46,XX, del(16p12.1-p11.2)**	**46,XX, del(16p12.1-p11.2)**	**46,XX, del(16p12.1-p11.2)**	NA
GM04592	47,XX,+21	**47,XX,+21**	**47,XX,+21**	**46,XX,+21**, del(1p31.1-p22.3)	−22
GM01359	47,XY,+18	**47,XY,+18**	**47,XY,+18**	**47,XY,+18**, dup(6q15-q16.1)	NA
GM03330	47,XY,+13	**47,XY,+13**	**47,XY,+13**	**47,XY,+13**	NA
GM01183	46,XY, der(4)t(4;12) (p16.1;p12)pat	**46,XY, del(4p16.3-p16.2), dup(12p13.33-p12.1)**	**46,XY, del(4p16.3-p16.2), dup(12p13.33-p12.1)**	**46,XY, del(4p16.3-p16.2), dup(12p13.33-p12.1)**	NA
Bulk	46,XX	**46,XX**	**46,XX**	**46,XX**	**46,XX**

Results consistent with the confirmed karyotypes were bolded.

## Discussion

In the present study, we examined the performance of four common WGA kits on Ion Proton sequencing platform. We extracted gDNA, single-cell, and multi-cell samples from six positive cell lines and humans’ oral epithelium, conducted whole genome amplification on them by MDA, GenomePlex, MALBAC, and PicoPLEX respectively, and sequenced them on Ion Proton platform. Then we estimated the quantity and size of WGA products, the quality, uniformity, reproducibility, and fidelity of sequencing data, and CNV detection performance of the four WGA kits. The results indicated that both MALBAC and PicoPLEX could yield high-quality data and showed high reproducibility and fidelity in reads distribution, but as for uniformity and genomic noise, according to MAPDs, PicoPLEX performed slightly better than MALBAC and closer to the distribution of the bulk-cell sample. Although GenomePlex had high uniformity, it contained excessive genomic noise, which had increased false positive errors in CNV detection. MDA showed the worst uniformity, reproducibility, and CNV detection performance among the four WGA kits, indicating that it was not suitable for CNV analysis.

In practical manufacture and application of WGA kits, there ought to be a reference standard in the kit. In theory, the reference standard could be a lysed single cell isolated from positive cell lines or corresponding DNA. Since DNA had an advantage in preservation and was more convenient in operation, we evaluated the property of the WGA products from gDNA sample, attempting to replace lysed cells with gDNA as reference standards. The MAPDs of the amplified gDNA samples were more than those of single-cell or multi-cell samples amplified by the same WGA kits, suggesting that there was more genomic noise in the amplified gDNA samples. The correlation coefficients between amplified gDNA samples and the bulk data was the lowest among the three sample types, which reflected the low fidelity in amplified gDNA samples. The bad performance of gDNA samples amplified by WGA kits might result from the nonrandom distributed DNA fragments in gDNA diluents.

Considering the relatively low throughput on Ion Proton, low-coverage whole genome sequencing was common on this platform but it was also a challenge for variant calling. To detect different variants, there were different choices of WGA kits. For small CNV detection, PicoPLEX and MALBAC were both good choices; for large CNV detection, PicoPLEX, MALBAC, and GenomePlex were competent; and for SNP calling, MDA and MALBAC were appropriate. Different projects focused on different variants. For example, preimplantation genetic screen currently focused on CNV detection [25–27], inheritable diseases studies were mainly interested in SNP calling [28–31], and cancer-related studies might concern CNVs, SNPs, and other variants [32,33]. There was no best method for all projects, but there was always a more appropriate one for a certain project.

In conclusion, we quantitatively examined the performance of four common commercial WGA kits on Ion Proton platform. Based on samples extracted from six positive cell lines and normal humans’ oral epithelium, we estimated the data yield, data quality, uniformity, reproducibility, fidelity, and CNV detection performance of PicoPLEX, GenomePlex, MALBAC, and MDA. At a similar yield and laboratorial situation, PicoPLEX showed the best performance in the quality of sequencing data, uniformity of read depth, amplification reproducibility and fidelity, so it was recommended for CNV calling on Ion Proton platform. Results in the present study would guide the selection of WGA kits on Ion Proton platform and assist the application of single-cell sequencing on this platform.

## Abbreviations

CNV: copy number variation
EDTA: ethylenediaminetetraacetic acid
gDNA: genomic DNA
LOESS: local regression, a non-parametric regression method based on k-nearest neighbors algorithm (k-NN)
MAPD: median absolute pairwise difference
MDA: multiple displacement amplification
NGS: next-generation sequencing
PBS: phosphate-buffered saline
SNP: single-nucleotide polymorphism
TBE: Tris-Borate-EDTA buffer
WGA: whole genome amplification
